# Series module of quinone-based organic supercapacitor (> 6 V) with practical cell structure

**DOI:** 10.1038/s41598-022-07853-6

**Published:** 2022-03-10

**Authors:** Yuto Katsuyama, Takayuki Takehi, Shu Sokabe, Mai Tanaka, Mizuki Ishizawa, Hiroya Abe, Masaru Watanabe, Itaru Honma, Yuta Nakayasu

**Affiliations:** 1 Department of Chemistry and Biochemistry, University of California Los Angeles, Los Angeles, 90095 USA; 2 Frontier Research Institute for Interdisciplinary Sciences (FRIS), Tohoku University, Sendai, 980-8578 Japan; 3 Division of General Education, National Institute of Technology Nagaoka College, Niigata, 940-0817 Japan; 4 Research Center of Supercritical Fluid Technology, Tohoku University, Sendai, 980-8579 Japan; 5 School of Engineering, Tohoku University, Sendai, 980-8579 Japan; 6 Institute of Multidisciplinary Research for Advanced Materials (IMRAM), Tohoku University, Sendai, 980-8577 Japan

**Keywords:** Batteries, Batteries

## Abstract

Inexpensive, high-performing, and environmentally friendly energy storage devices are required for smart grids that efficiently utilize renewable energy. Energy storage devices consisting of organic active materials are promising because organic materials, especially quinones, are ubiquitous and usually do not require harsh conditions for synthesis, releasing less CO_2_ during mass production. Although fundamental research-scale aqueous quinone-based organic supercapacitors have shown excellent energy storage performance, no practical research has been conducted. In this study, we aimed to develop a practical-scale aqueous-quinone-based organic supercapacitor. By connecting 12 cells of size 10 cm × 10 cm × 0.5 cm each in series, we fabricated a high-voltage (> 6 V) aqueous organic supercapacitor that can charge a smartphone at a 1 C rate. This is the first step in commercializing aqueous organic supercapacitors that could solve environmental problems, such as high CO_2_ emissions, air pollution by toxic metals, and limited electricity generation by renewable resources.

## Introduction

Renewable energy generated approximately 21% of all the electricity in the United States in 2020, the second-largest power generation following natural gas^1^. Although this is an astonishing announcement, further energy shifts toward power generation from renewable resources are required to reduce CO_2_ emissions and mitigate climate change^2,3^. One effective strategy to use renewable energy efficiently is to introduce smart grids, which require a large number of stationary energy storage devices^2,4,5^. However, the inorganic batteries currently in use are expensive because they use rare metals that are limited resources and are unevenly distributed, sometimes available only in conflict zones^6–8^. In addition, inorganic materials are often synthesized under harsh conditions, such as high temperature and pressure, which consumes a large amount of energy^7,9^. To make matters worse, the use of toxic metals, such as cobalt, induces severe environmental pollution^7,10,11^. Therefore, it would be a better choice to avoid using such inorganic batteries for smart grids.

Organic batteries, the active materials of which are organic compounds, can potentially solve such environmental issues. Firstly, organic energy storage devices consist of ubiquitous light elements such as carbon, oxygen, nitrogen, sulfur, and hydrogen. Therefore, they have almost no restrictions on resource availability^10,12–14^. Secondly, organic compounds are often synthesized under mild conditions from renewable resources, which does not require a huge amount of energy for mass production as inorganic materials do^7,10,11,14–16^. Thirdly, it is easy to tune the theoretical capacity and redox potential by modifying the molecular structure^10,14,17^. Carbonyl compounds, especially quinones, have two redox centers in a single molecule, leading to a high capacity of up to 496 mAh g^–1^. Their redox potentials are adjustable in the range of 1.7–3.2 V *vs*. Li/Li^+^ by molecular engineering^18,19^. In addition, such small organic molecules have advantages over other polymer-based organic active materials because their production process can be more straightforward and inexpensive. Furthermore, the formation of conjugated polymers fundamentally lowers both redox potentials and theoretical capacities. The general challenges encountered by small organic molecules are their low electric conductivity and intensive dissolution into the electrolyte^20–24^. Several approaches have been proposed to overcome these issues^25–30^. One effective method is to impregnate quinones in the micropores of porous carbon materials to provide conductive paths and suppress the dissolution of quinones into the electrolyte^21,23,24,26^. By using this strategy, a full-cell redox supercapacitor with a tetrachlorohydroquinone (TCHQ) cathode and a dichloroanthraquinone (DCAQ) anode was proposed in 2014, showing an energy density of ~ 14 Wh kg^–1^ with excellent rate performance and no capacity loss even after 10,000 cycles^23^.

Although the aqueous quinone supercapacitor has shown satisfactory performances at the fundamental research scale (electrode mass, diameter, and thickness each less than 6 mg, 7 mm, and ~100 µm, respectively), some obstacles must be overcome when considering its practical applications. For instance, it should be verified that the practical-size electrodes with sufficient mass loadings still show capacities and voltages comparable to those of the fundamental research-scale electrodes. Another problem is that this type of redox supercapacitor uses acidic aqueous electrolytes (e.g., 0.5 M H_2_SO_4_ aq.), which requires the use of acid-durable materials for cell components. At the fundamental research scale, a beaker and gold mesh are used for the cell container and current collector, respectively, which are strong against acidic solutions. However, beaker cells are made of glass and can be easily broken by an external shock, which is dangerous for practical applications. In addition, gold-mesh current collectors inflate cell costs. Therefore, these materials must be replaced by other inexpensive, acid-durable materials to address safety and cost issues.

This study is the first to develop quinone-based aqueous supercapacitors with a practical cell structure (10 cm × 10 cm × 0.52 cm, 1 g/electrode) that can charge a smartphone. We studied alternative materials for cell containers and current collectors, as well as cell configurations, and proposed one possible solution. Finally, we fabricated a high-voltage aqueous supercapacitor up to 7.2 V by connecting twelve cells in series. We believe that the knowledge obtained from this study will promote research on practical applications of aqueous organic supercapacitors, which has been lacking but is essential and must be tackled simultaneously with fundamental research.

## Results and discussion

### Cell configuration

First, we used austenitic stainless steel (SUS316), which is stable in diluted sulfuric acid at room temperature, to replace the gold-mesh current collector^31^. Figure S1 shows the charge and discharge curves of the DCAQ electrodes when Au and SUS316 were used as the current collectors. The SUS316 mesh current collector exhibited no capacity loss compared to a gold mesh, showing a similar charge/discharge curve. However, the discharge curve of SUS316 has a ~ 20 mV larger overpotential in the plateau region compared to that of the gold mesh, and the curve is gentler in the slope region. The difference in overpotential can be attributed to the different electrical conductivities of gold and SUS316. The gentler slope of SUS316 could stem from the side reactions to form/destroy the passivation layer on SUS316^32^. Only such slight differences were observed even when an expensive gold-mesh current collector was replaced with an inexpensive SUS316 current collector, suggesting that SUS316 is a good alternative in terms of both the cost and performance. Based on the above considerations, we decided to use a SUS316 mesh as the current collector and a SUS316 plate as the cell packaging material for acidic aqueous quinone-based supercapacitors. For clarity, changes in the cell components from the fundamental research scale to the practical scale are summarized in Table 1.

**Table 1:** Changes of the cell components from the fundamental research scale to the practical scale.

Component	Fundamental level	Practical level
Current collector	Gold mesh	Austenitic stainless steel (SUS316) mesh
Cell container	Glass	SUS316
Electrode thickness	~ 100 um	~ 1.0 mm
Electrode mass	< 6 mg	1 g
Electrode size	7 mm diameter (0.38 cm^2^)	15–20 cm^2^

**Figure 1:**
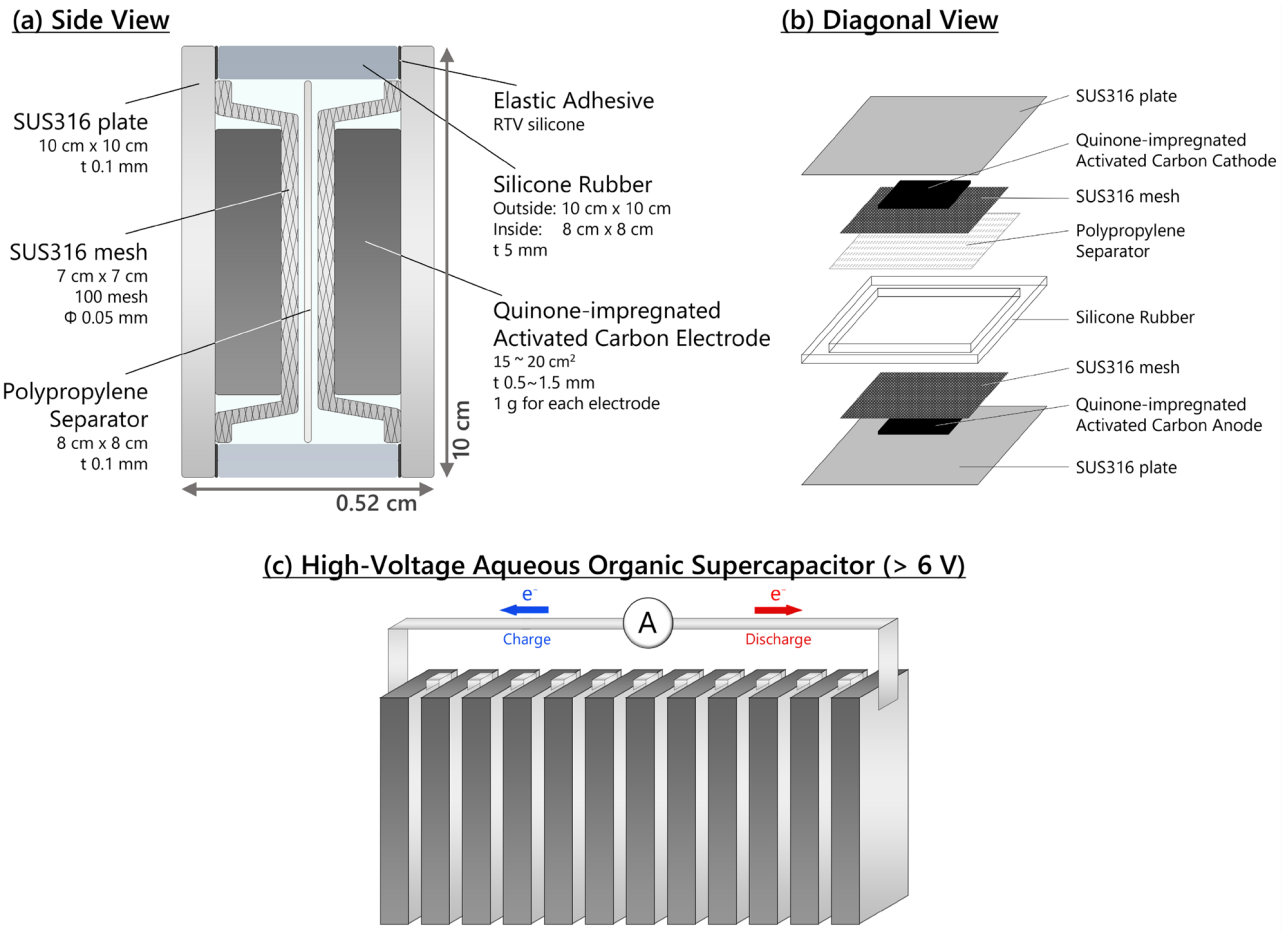
Illustrations of the cell configuration of a 10 cm × 10 cm × 0.52 cm single cell from (**a**) side view and (**b**) diagonal view. (**c**) A high-voltage aqueous organic supercapacitor (> 6 V) by connecting twelve single cells in series.

The single-cell configuration is shown in Fig. 1a,b. A 0.5–1.5 mm thick quinone-impregnated activated carbon electrode weighing 1 g is pressed on a SUS316 mesh current collector. The collector is welded to the SUS316 plate so that the mesh and plate sandwich the quinone-containing electrode. The sandwich structure prevents the electrode from peeling off from the current collector. The two electrodes were separated using a polypropylene separator. Silicone rubber was used as a gasket and was adhered to the SUS316 plate using RTV silicone. The cell was filled with a 0.5 M H_2_SO_4_ aqueous electrolyte. The size of a fabricated single cell was 10 cm × 10 cm × 0.52 cm. We prepared a high-voltage aqueous organic supercapacitor by connecting 12 single cells in series (Fig. 1c). Electrodes with 1.0 mm thickness are used for a high-voltage supercapacitor.

### Battery performance of a single cell (10 cm × 10 cm × 0.52 cm)

The battery performances of the practical-size electrodes (e.g., 1 mm thick and 1 g weight) were tested. Firstly, the utilization rate of a chloranil-impregnated activated carbon electrode with a thickness of 0.5 mm was determined by a half-cell test. Figure S2 shows the galvanostatic discharge curve of the electrode with a redox capacity of 161 mAh g^–1^, which was calculated by subtracting the electric double-layer (EDL) capacity from the total capacity. The utilization rate, defined as the ratio of the experimentally obtained redox capacity to the theoretical redox capacity (chloranil: 217.9 mAh g^–1^), was 73.8%. Although the 0.5 mm thick electrode has more than five times higher mass loading than the fundamental research-scale electrode, the utilization rate is comparable with those of the fundamental research-scale electrodes (75–86%)^23,24^. This could be attributed to the fast kinetics of proton diffusion, which has the highest diffusion coefficient known to date, through the thick electrode^33^.

**Figure 2:**
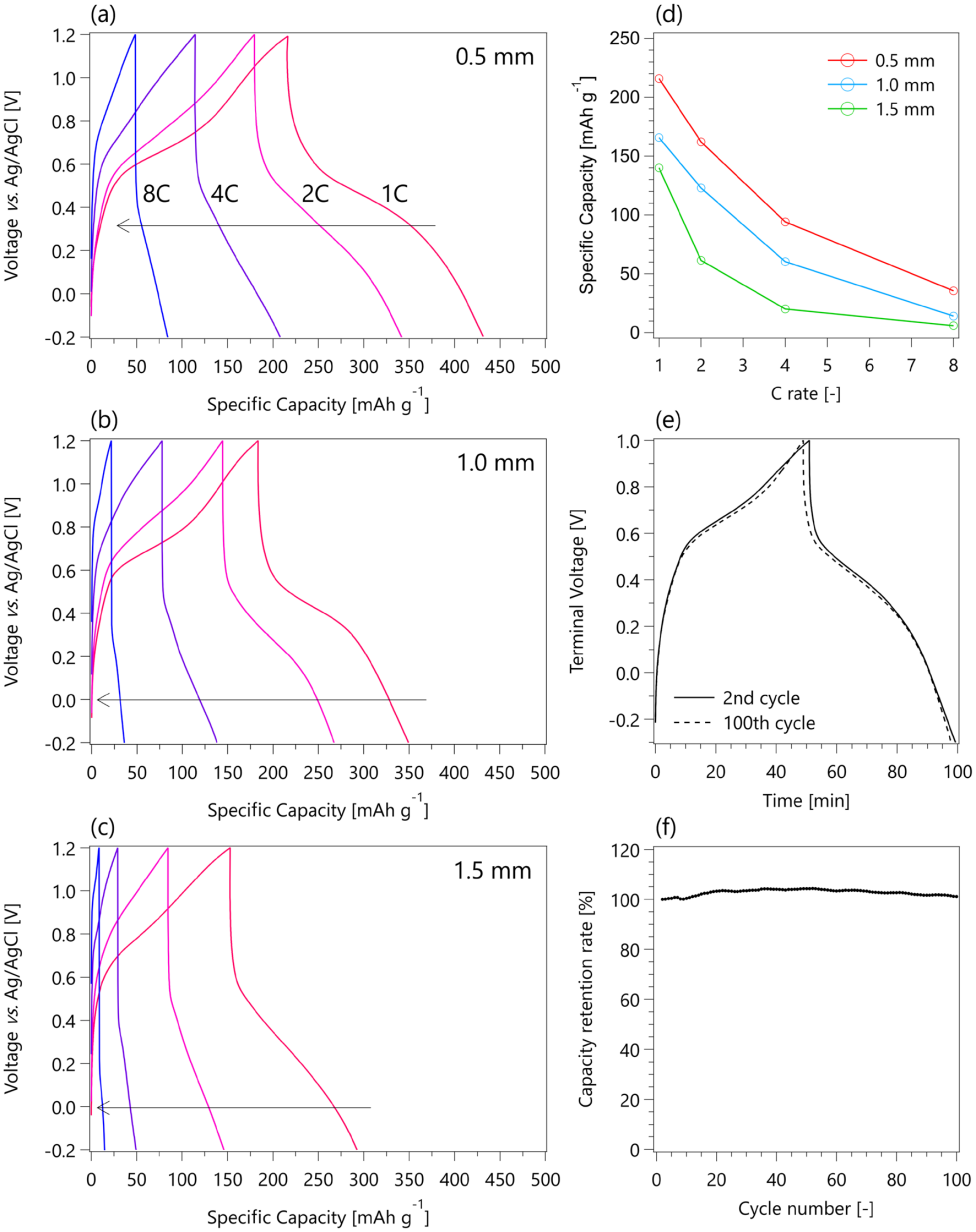
(**a**–**c**) Rate performances of 10 cm × 10 cm × 0.52 cm single cells with different electrode thicknesses (0.5, 1.0 and 1.5 mm). (**d**) The specific capacities of the three types of single cells (0.5, 1.0 and 1.5 mm) at 1, 2, 4, and 8 C rates. (**e**) Charge and discharge profiles of a single cell at a 1 C rate with 1.0 mm thickness at the 2nd and the 100th cycles. (**f**) The capacity retention rate of a single cell at a 1 C rate for 100 cycles.

A full cell was fabricated by combining a chloranil cathode (1 g) and a DCAQ anode (1 g) to form a 10 cm × 10 cm × 0.52 cm single cell, as shown in Fig. 1a,b. The charge and discharge profiles of the 0.5, 1.0, and 1.5 mm thick electrodes at 1, 2, 4, and 8 C rates are displayed in Fig. 2a–c, and their rate performances are summarized in Fig. 2d. Although a full cell with 0.5 mm thick electrodes exhibited a high capacity of 216 mAh g^–1^ at a 1 C rate, the full cell with 1.0 and 1.5 mm thick electrodes retained only 166 and 140 mAh g^–1^, respectively. This result clearly shows the tendency of the capacity to decrease as the mass loading increases. Figure S3 shows the capacity retention rates of the three types of electrodes at 2, 4, and 8 C rates based on the capacity at a 1 C rate. At a 2 C rate, full cells with 0.5 and 1.0 mm electrodes retained approximately 75% of their capacities, while the full cell with 1.5 mm electrodes retained only 44%. When the charge/discharge rates were further increased to 4 C, the full cells with 0.5, 1.0, and 1.5 mm electrodes maintained 44%, 36%, and 14% of the capacities, respectively. At 8 C, only 16%, 8.5%, and 4.3% of the capacities were retained. Considering that fundamental research-scale electrodes retain more than 80% of their capacities at a high rate (~ 5 C), making the electrodes thicker critically impairs their superb rate performances. This is because the diffusion length of protons becomes longer in the thicker electrodes, and  the redox reactions of quinones cannot proceed above a certain rate due to an insufficient supply of protons. However, state-of-the-art technologies introducing macropores into the thick electrodes can dramatically improve the sluggish ion transfer inside the thick electrodes^34–38^. The energy density of the single cell with 0.5 mm thick electrodes was 10.9 Wh kg_total electrode_^–1^ and 40.3 Wh kg_active material_^–1^ at a 1 C rate, which was 87.2% of the energy density at the fundamental research scale (12.5 Wh kg_total electrode_^–1^)^24^. This high energy density again confirmed that this practical-scale redox supercapacitor with thick electrodes can be useful even at a 1 C rate. The slight loss of energy density can be attributed to the lower utilization rate of organic active materials, as discussed above. In addition, the chloranil cathode, which has a larger theoretical capacity than the DCAQ anode, could not use all its capacity because the weights of the cathode and anode were identical. Figure 2e shows the charge and discharge profiles of the single cell at the 2nd and 100th cycles at a 1 C rate. There were almost no differences between the two curves, indicating that no capacity loss was observed over 100 cycles. Figure 2f shows the capacity retention rate of the single cell for the first 100 cycles at a rate of 1 C, showing that 101.1% of the capacity was retained after 100 cycles. An excellent cycle performance was maintained even when the cell was scaled up to a practical size^23^.

### Battery performance of a high-voltage cell (12 single cells are connected in series)

**Figure 3:**
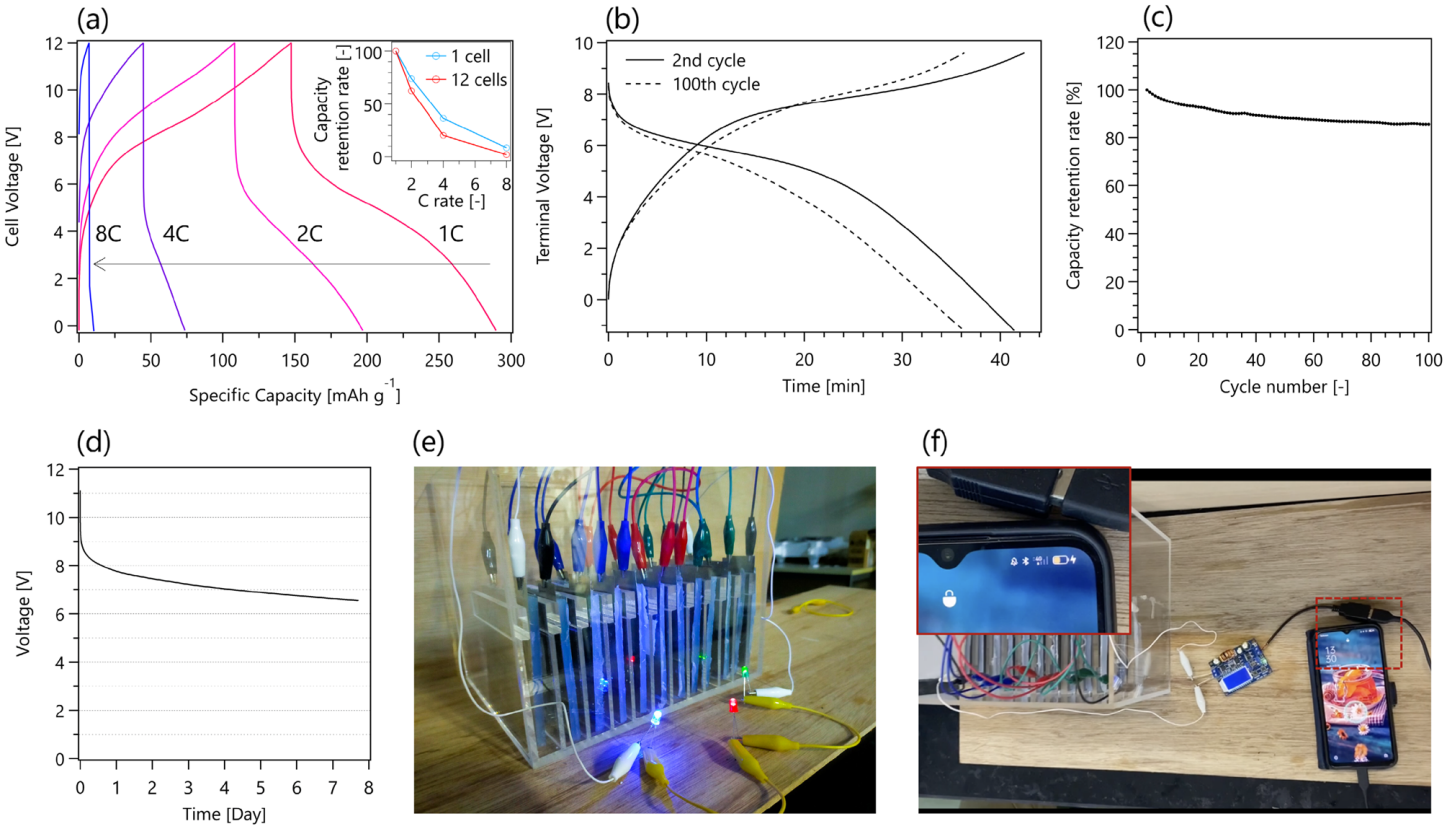
(**a**) Charge and discharge profiles of a high-voltage cell (twelve single cells connected in series) with different C rates (1 C, 2 C, 4 C, and 8 C). The inset shows the capacity retention rates of a single cell and a high-voltage cell based on the capacities at a 1 C rate. (**b**) Charge and discharge profiles of a high-voltage cell at a 1 C rate for the 2nd and 100th cycle. (**c**) The capacity retention rate of a high-voltage cell for 100 cycles at a 1 C rate. (**d**) Transition of open-circuit voltage of a fully charged high-voltage cell for more than 7 days. (**e**) A picture of a high-voltage aqueous supercapacitor illuminating three different LED bulbs (red, green, and blue) connected in series, which requires at least 6.9 V. (**f**) A smartphone is charged by a high-voltage aqueous supercapacitor.

As shown in Fig. 3e, a high-voltage aqueous supercapacitor was fabricated by connecting 12 single cells in series using coated copper wires. The device successfully illuminated three different LED bulbs (red, green, and blue) connected in series, which required at least 6.9 V. Figure 3f demonstrates that a smartphone can be charged using a high-voltage cell via a current regulator.

Figure 3a shows the charge and discharge curves of the high-voltage cell at rates of 1, 2, 4, and 8 C. The average discharge voltage of a high-voltage cell at a 1 C rate was 4.39 V, which is almost equivalent to the average discharge voltage of a single cell multiplied by 12 (0.387 V × 12 cells = 4.65 V). Therefore, the total overpotential induced by the series connection was only 0.26 V. The specific discharge capacity was 142 mAh g^–1^ at a rate of 1 C, which is 86% of the capacity of a single cell. The inset of Fig. 3a shows the capacity retention rates at different C rates. A high-voltage cell has a slightly lower capacity retention rate than a single cell: 62.6% is retained at 2 C; 20.3% at 4 C; and 8.5% at 8 C. The energy density of the high-voltage cell at a rate of 1 C is 7.1 Wh kg_total electrode_^–1^, which is lower than that of a single cell. When cells are connected in series, the overall capacity is determined by one single cell, which has the lowest capacity among all connected cells. Furthermore, all single cells must have similar voltages to utilize them efficiently. If each single cell has a different voltage, it is impossible to fully charge and discharge all the cells. Therefore, the slightly lower capacity and inferior rate performance of the high-voltage cell can be attributed to the slight differences in capacity and voltage of each of the 12 cells connected in series.

The cycle performance of the high-voltage cell is shown in Fig. 3b,c. From the discharge curves at the 2nd and 100th cycles, we found that the plateau capacity decreased after 100 cycles, and the capacity retention rate at the 100th cycle was 85%. Therefore, the primary degradation mechanism of a high-voltage cell is the decreased redox reactions of quinones. This result differs from that of a single cell with excellent cyclability. The slight voltage difference of each single cell would be the reason why the cycle performance deteriorates when twelve single cells are connected in series. The slight voltage difference could cause some single cells to be overcharged or overdischarged, producing gases at the electrode by electrochemical decomposition of the aqueous electrolyte. The area of the electrode in contact with the generated gases becomes electrochemically inactive and therefore can no longer undergo redox reactions. To avoid this issue, a voltage regulator that can control the voltage of each cell is required. Figure 3d shows the transition of the open-circuit voltage of a fully charged high-voltage cell over 7 days. Even after 7 days, the open-circuit voltage remains at 6.6 V, which is higher than the discharge plateau voltage. This implies that the voltage loss is caused only by the destruction of the EDL, and most quinones are stable in the charged states and do not degrade even after 7 days.

## Conclusion

To the best of our knowledge, this is the first study to scale up a quinone-based aqueous organic supercapacitor to a practical scale that is safe, inexpensive, and environmentally benign. To increase the safety and reduce the cost, glass cell containers and gold current collectors, which have been used for fundamental research, have been successfully replaced by sulfuric acid-resistant austenitic stainless steel (SUS316) without sacrificing energy storage performances^31^. To increase the total capacity of a single cell, the electrode thickness was scaled up from the fundamental research scale (~ 100 µm) to the practical scale (0.5–1.5 mm). The energy density of a practical-scale single cell with 0.5 mm thick electrodes was 10.9 $\text{Wh} \; {\text{kg}}_{\text{total} \; \text{electrode}}{^{-1}}$ and 40.3 $\text{Wh} \; {\text{kg}}_{\text{active} \; \text{material}}{^{-1}}$ at a 1 C rate, which is 87.2% of the energy density at the fundamental research scale (12.5 $\text{Wh} \; {\text{kg}}_{\text{total} \; \text{electrode}}{^{-1}}$). This confirms that a sufficient energy density is maintained even when 0.5 mm thick electrodes are used^24^. The cyclability of a single cell was excellent, in that 101.1% was retained after 100 cycles at a 1 C rate. However, when the charge/discharge rate was increased, the capacity dropped faster than the fundamental research-scale electrodes. This is because the diffusion length of protons becomes longer in the thicker electrodes, and the redox reactions of quinones cannot proceed above a certain rate due to an insufficient supply of protons. By connecting twelve single cells in series, we prepared a high-voltage supercapacitor (> 6 V) capable of illuminating three different LED bulbs (red, green, and blue) connected in series, which requires at least 6.9 V, and charging a smartphone. The high-voltage cell showed a slightly lower energy density and cycle performance than the single cell. This could be attributed to the differences in capacity and voltage of each of the 12 single cells connected in series, which could be solved by introducing a voltage regulator that can control the voltage of each single cell. Through this study, we have taken the first step toward the practical application of safe, inexpensive, and environmentally benign quinone-based aqueous supercapacitors, which can facilitate the introduction of smart energy grids and enable more efficient use of renewable energy.

## Methods

### Materials preparation

Quinones (chloranil and dichloroanthraquinone (DCAQ)) were purchased from Tokyo Chemical Industry Co., Ltd., and activated carbon (MAXSORB) was purchased from Kansai Coke and Chemicals Co., Ltd. We purchased chloranil instead of TCHQ because chloranil is more stable in air than tetrachlorohydroquinone (TCHQ) and is hence more inexpensive. They were used as received without further purification. Active materials impregnated with chloranil (3 g) and DCAQ (3 g) were dissolved in 1.5 and 3 L of acetone, respectively, and sonicated for 10 min. Activated carbon (7 g) was dispersed in the solution via sonication for 1 h. The solution was stirred at 80 °C to evaporate acetone, and quinones were impregnated into the nanopores of the activated carbon. The obtained powder was mixed with a polytetrafluoroethylene (PTFE) binder at a weight ratio of 9:1, and the pellet was then formed. Notably, a conductive additive was not used. Therefore, the weight ratio of the electrode is as follows; quinone: activated carbon: PTFE = 27:63:10.

### Fabrication of a single cell and a high-voltage cell

The quinone-impregnated activated carbon pellet electrode was pressed on a SUS316 mesh (7 cm × 7 cm, 100 mesh, Φ 0.05 mm) at 4 kN. The pressed pellet was stretched to different thicknesses (0.5, 1, and 1.5 mm) by a heated draw roller at 60 °C. The footprint area of the electrode was approximately 15–20 cm^2^. The chloranil electrode was reduced to TCHQ at a 1.3 C rate in a half-cell configuration: Ag/AgCl (3 M NaCl aq.) was used as the reference electrode, and an excess amount of activated carbon electrode (activated carbon: PTFE = 9:1) was used as the counter electrode. Each pellet adhered to the mesh was welded to the SUS316 plate (10 cm × 10 cm, 0.1 mm thickness) so that the electrode was sandwiched between the mesh and the plate. The electrodes were soaked in the electrolyte (0.5 M H_2_SO_4_ aqueous solution) and vacuumed for one hour to remove the gas from the pores of the electrode. A polypropylene separator (Nippon Kodoshi Co.) was used. A 5 mm thick silicone rubber (Togawa Rubber Co. Ltd.) was shaped by a cutter to be 10 cm × 10 cm outside and 8 cm × 8 cm inside. This was used as a gasket and was adhered to the SUS316 plates using an RTV silicone (Wako Chemical, Ltd.). The weights of the cathode and anode were identical. The cell was filled with a 0.5 M H_2_SO_4_ aqueous electrolyte. A high-voltage cell was prepared by connecting 12 single cells in series using coated copper wires.

### Electrochemical test

To electrochemically reduce chloranil to TCHQ before fabricating a full cell, a potentiostat (HSV-110) and power supplier (Kikusui Electronics Corp.) were used. A potentiostat (Solartron Analytical) was used for the other electrochemical measurements. The voltage range for the charging and discharging test was – 0.2 V to 1.2 V for a single cell, and – 0.2 V to 12.0 V for a full cell. For full-cell supercapacitors, C rates were calculated based on the theoretical capacity of the chloranil cathode. Energy density was calculated from the total mass of both electrodes or the total mass of the active materials in both electrodes.

## Supplementary Information

Supplementary Figures.
