# Supplementary material for: Series module of quinone-based organic supercapacitor (> 6 V) with practical cell structure
Source: Sci Rep. 2022 Mar 10;12:3915. doi: 10.1038/s41598-022-07853-6 (PMC8913612; doi:10.1038/s41598-022-07853-6)
Supplement: Supplementary file 1 — Supplementary Figures. [file 41598_2022_7853_MOESM1_ESM.docx]

Supporting Information

Quinone-based organic supercapacitor (> 6 V) with practical series-module cell structure

# Yuto Katsuyama,^1,2^ Takayuki Takehi,^2,3^ Shu Sokabe,^4^ Mai Tanaka,^5^ Mizuki Ishizawa,^5^ Hiroya Abe,^2^ Masaru Watanabe,^4^ Itaru Honma^6^ and Yuta Nakayasu*^2,4^

1Department of chemistry and Biochemistry, University of California Los Angeles, Los Angeles, 90095, USA
2Frontier Research Institute for Interdisciplinary Sciences (FRIS), Tohoku University, Sendai, 980-8578, Japan
3Division of General Education, National Institute of Technology Nagaoka College, Niigata, 640-8532, Japan
4Research Center of Supercritical Fluid Technology, Tohoku University, Sendai, 980-8579, Japan
5School of Engineering, Tohoku University, Sendai, 980-8579, Japan
6Institute of Multidisciplinary Research for Advanced Materials (IMRAM), Tohoku University, Sendai, 980-8577, Japan

*[nakayasu@tohoku.ac.jp](mailto:corresponding.author@email.example)

**S1. SUS vs. Au**

Figure S1 (a) shows the charge and discharge curves of the DCAQ electrodes when Au and SUS316 were used as the current collectors. The charge and discharge curves at different C rates (1 and 0.5 C) are shown in Fig. S1 (b). A discussion is provided in the main manuscript.


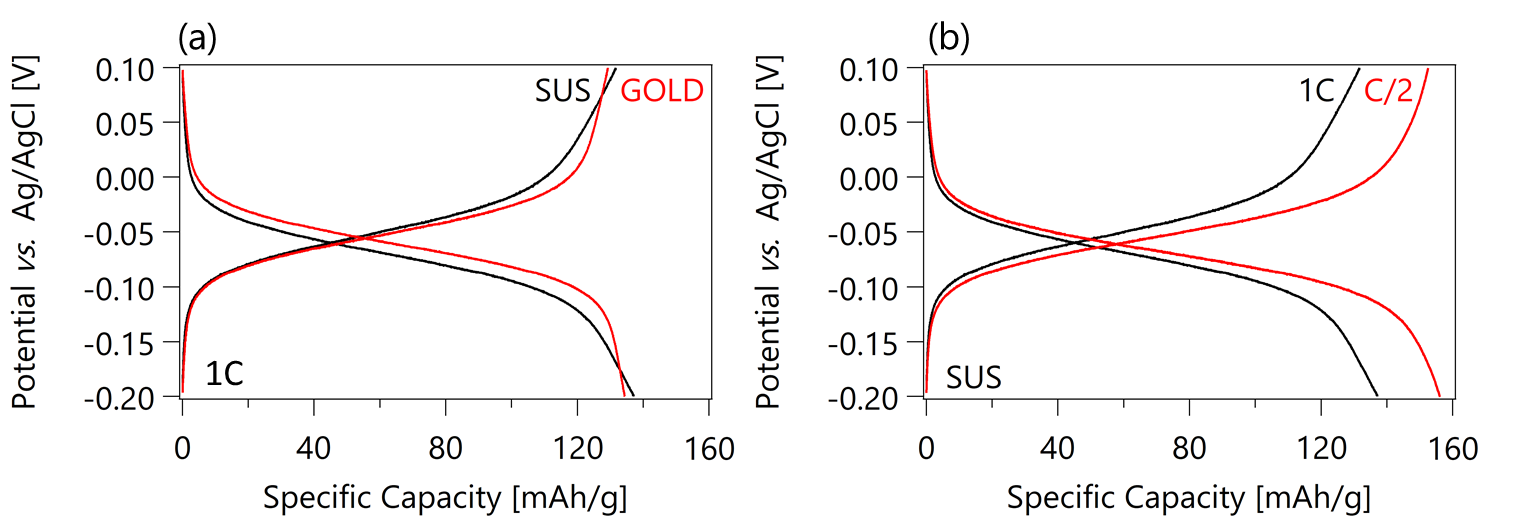


**Figure S1**. (a) Charge and discharge curves of DCAQ electrodes when Au and SUS316 are used as current collectors at a 1 C rate. (b) Charge and discharge curves of DCAQ electrodes when SUS316 mesh is used as current collector at different C rates (1 and 0.5 C)

**S2. Calculation of redox capacity**

The capacity obtained by redox reactions of quinones is calculated as follows:

1. Draw a linear trend line in the voltage region that is completely dominated by the formation/destruction of the electric double layers (EDL).

2. The intersection of the trend line and termination voltage must be the capacity obtained by the EDL (20.4 mAh g^–1^).

3. By subtracting the EDL capacity from the total capacity, the redox capacity can be calculated (181.3 – 20.4 = 160.9 mAh g^–1^).


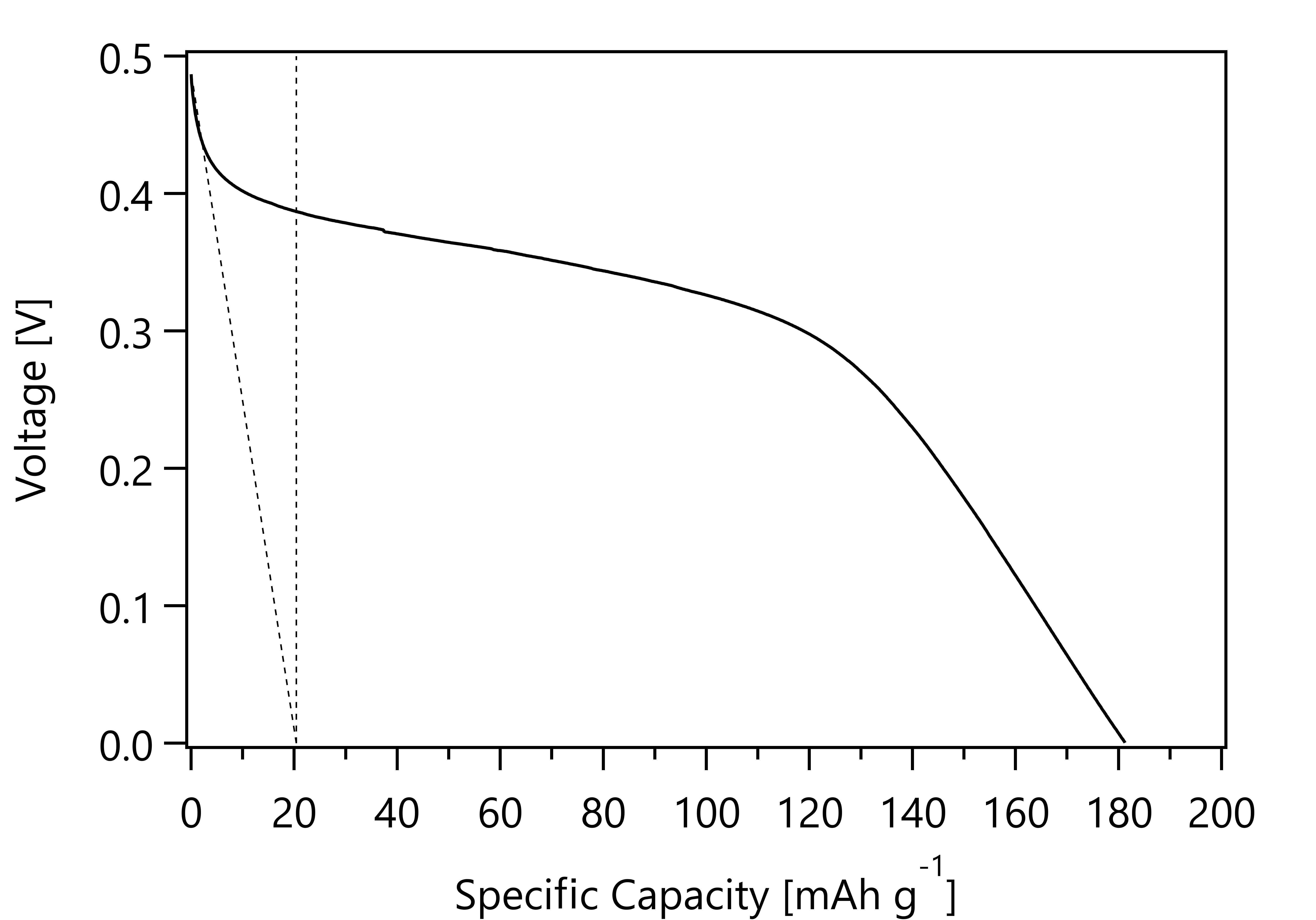


**Figure S2**. Discharge curve of a chloranil electrode with a thickness of 0.5 mm at a 1 C rate.

**S3. Rate performance of thick electrodes**

Figure S3 is another expression of Figure 2 (d), representing the capacity retention rates of single cells with different electrode thicknesses at high C rates based on the capacities at a 1 C rate. As the electrode became thicker, the capacity decreased faster at higher C rates.


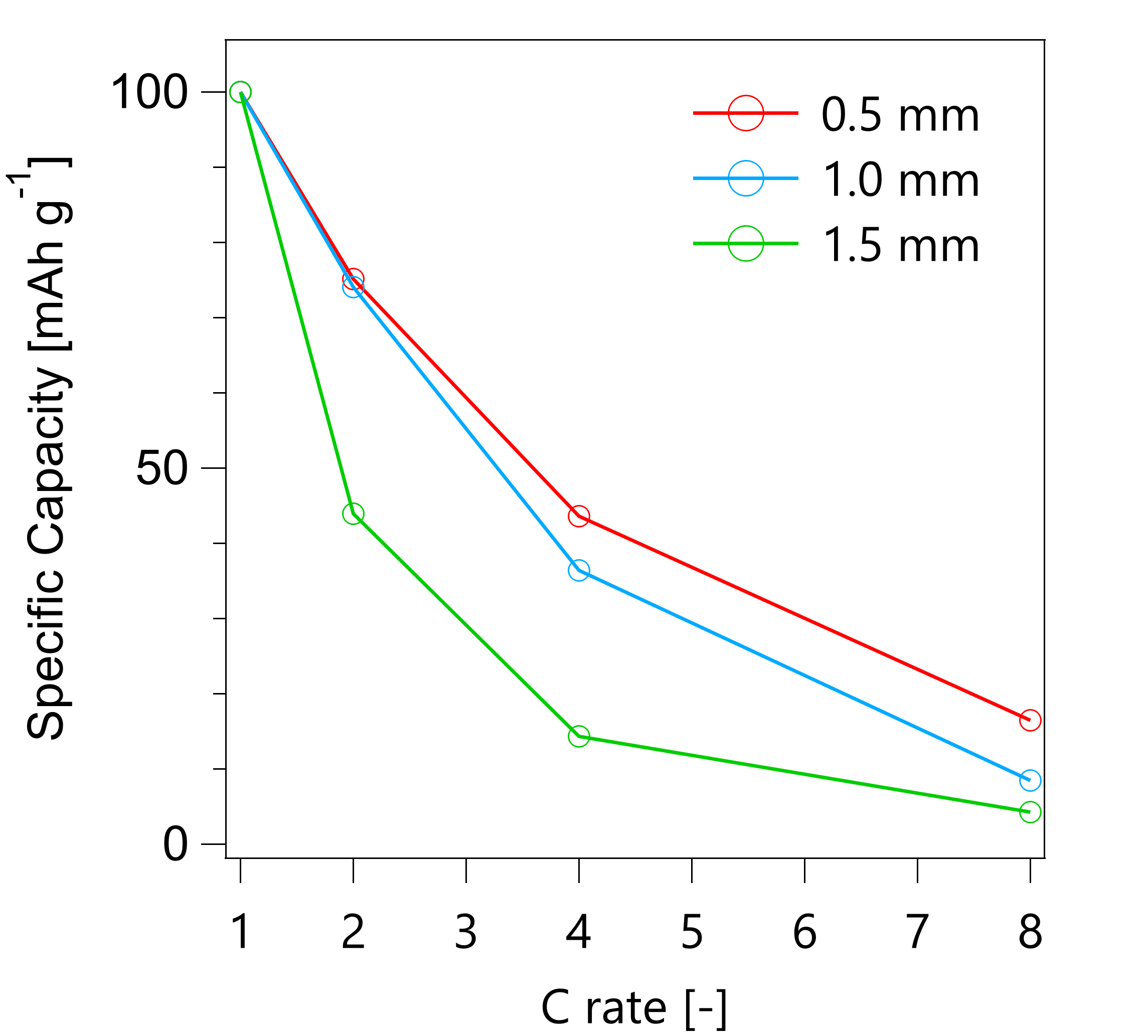


**Figure S3**. Capacity retention rates of single cells with different electrode thicknesses (0.5, 1.0, and 1.5 mm) at high C rates based on the capacities at a 1 C rate.

**S4. pH dependency**

From the standpoint of safety, it is better to use a neutral electrolyte than an acidic electrolyte when the device is practically used. Cyclic voltammetry curves of the chloranil and DCAQ electrodes in H_2_SO_4_ aqueous electrolyte at various pH values (0, 3, and 4) are shown in Figure S4. Both the chloranil and DCAQ electrodes exhibit sharp redox peaks only at pH=0, suggesting that the supercapacitor works well only under strongly acidic conditions.


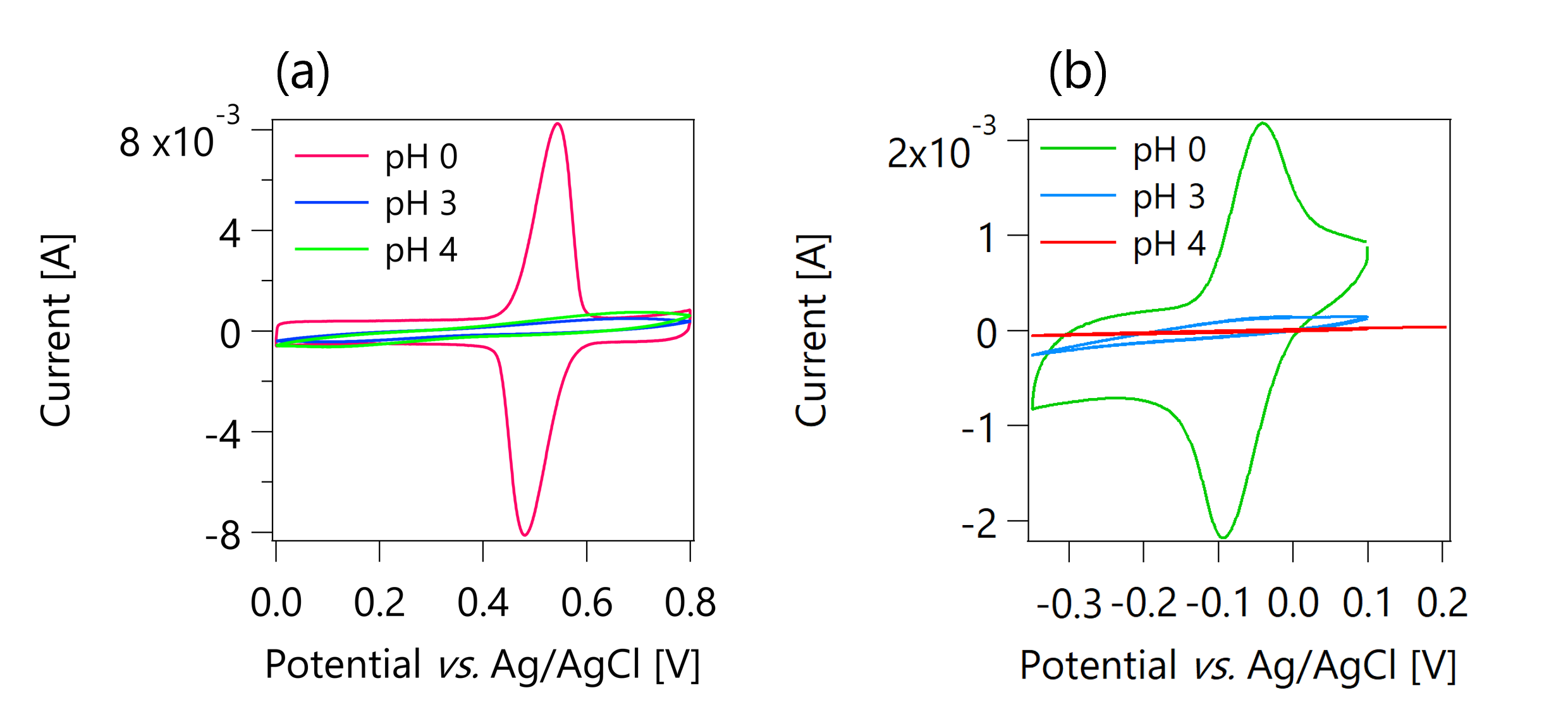


**Figure S4**. Cyclic voltammetry curves of (a) chloranil and (b) DCAQ electrodes in H_2_SO_4_ aqueous electrolytes with various pH values (0, 3, and 4).
